# Supplementary material for: Microbiota-accessible fiber activates short-chain fatty acid and bile acid metabolism to improve intestinal mucus barrier in broiler chickens
Source: Microbiol Spectr. 2023 Dec 14;12(1):e02065-23. doi: 10.1128/spectrum.02065-23 (PMC10782983; doi:10.1128/spectrum.02065-23)
Supplement: Supplemental material — Tables S1 and S2; Fig. S1 to S3. [file spectrum.02065-23-s0001.pdf]

Table S1. Formulation and nutrient composition of experimental diets (as-fed basis, %)

Ingredients, %	NF	Control	β-glucan	Arabinoxylan	Resistant starch
Corn	57.27	0.00	0.00	0.00	0.00
Corn starch	0.00	58.25	55.25	55.25	55.25
Soybean meal	35.2	0.00	0.00	0.00	0.00
Soy protein concentrate	0.00	32.68	32.68	32.68	32.68
Cottonseed meal	2.00	0.00	0.00	0.00	0.00
Soybean oil	2.00	3.52	3.52	3.52	3.52
Sucrose	0.00	0.50	0.50	0.50	0.50
β-glucan	0.00	0.00	3.00	0.00	0.00
Arabinoxylan	0.00	0.00	0.00	3.00	0.00
Resistant starch	0.00	0.00	0.00	0.00	3.00
NaCl	0.36	0.31	0.31	0.31	0.31
Limestone	2.00	0.80	0.80	0.80	0.80
Calcium hydrogen phosphate	0.30	3.32	3.32	3.32	3.32
Choline chloride	0.05	0.10	0.10	0.10	0.10
L-lysine hydrochloride	0.14	0.00	0.00	0.00	0.00
Mineral premix[a]	0.30	0.10	0.10	0.10	0.10
Phytase	0.10	0.00	0.00	0.00	0.00
Vitamin premix[b]	0.03	0.03	0.03	0.03	0.03
DL-methionine	0.25	0.16	0.16	0.16	0.16
Sodium bicarbonate	0.00	0.20	0.20	0.20	0.20
Antioxidant	0.00	0.03	0.03	0.03	0.03
Total	100	100	100	100	100
Calculated nutrient level					
Metabolic energy, kcal/kg	2950	2960	2866	2866	2866
Dry matter	86.41	95	95	95	95
Crude fiber	3.02	1.67	1.67	1.67	1.67
Crude protein	21.91	21.00	21.00	21.00	21.00

Ash	6.26	6.42	6.42	6.42	6.42
Total phosphorus	0.61	0.61	0.61	0.61	0.61
Calcium	0.96	0.96	0.96	0.96	0.96
Methionine	0.57	0.45	0.45	0.45	0.45
Lysine	1.21	1.38	1.38	1.38	1.38

[a]Mineral premix provided the following per kg of the diet: Mn, 80 mg; I, 0.40 mg; Fe, 80 mg; Cu, 10 mg; Zn, 70 mg; Se, 0.30 mg

[b]Vitamin premix provided the following per kg of the diet: vitamin A, 250,000 IU; vitamin D, 50,000 IU; vitamin K3, 53 mg; vitamin B1, 40 mg; vitamin B2, 120 mg; vitamin B12, 0.50 mg; vitamin E, 600 IU; biotin, 0.65 mg; folic acid, 25 mg; pantothenic acid, 240 mg; niacin, 1,000 mg.

Table S2. Primer sequences used for Real-time quantitative PCR

Genes	Accession numbers	Forward Primer	Reverse Primer
β-actin	NM_205518.1	ATTGTCCACGCAAATGCTTC	AAATAAAGCCATGCCAACTCGTC
MUC2	NM_001318434.1	TTCATGATGCCTGCTCTTGTG	CCTGAGCCTTGGTACATTCTTG
ZO-1	XM_015278981.2	GGGATGTTTATTTGGGCGGC	TCACCGTGTGTTGTTCCCAT
Occludin	NM_205128.1	TCATCGCCTCCATCGTCTAC	TCTTACTGCGCGTCTTCTGG
Claudin	NM_001013611.2	ACCCACAGCCTAAGTGCTTC	AGGTCTCATAAGGCCCCACT

Abbreviations: *β-actin*, the internal reference gene beta actin, *MUC2,* mucin 2; *ZO-1*, zonula occludens 1.

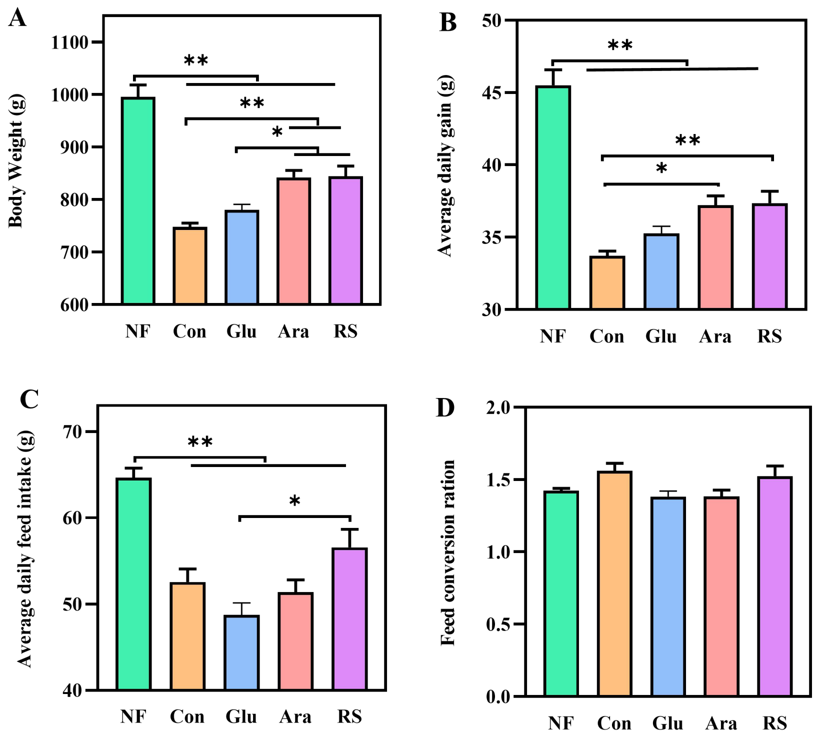

Figure S1. Effects of β-glucan, arabinoxylan or resistant starch supplementation on growth performance in broilers. (A) Body weight, (B) Average daily gain, (C) Average daily feed intake, (D) Feed conversion ration. All data are expressed as mean ± SEM (n=10). * means $P < 0.05$, ** means $P < 0.01$. NF, norml fiber levels groups; Con, dietary fiber deprivation group; Glu, β-glucan group; Ara, arabinoxylan group; RS, resistant starch group.

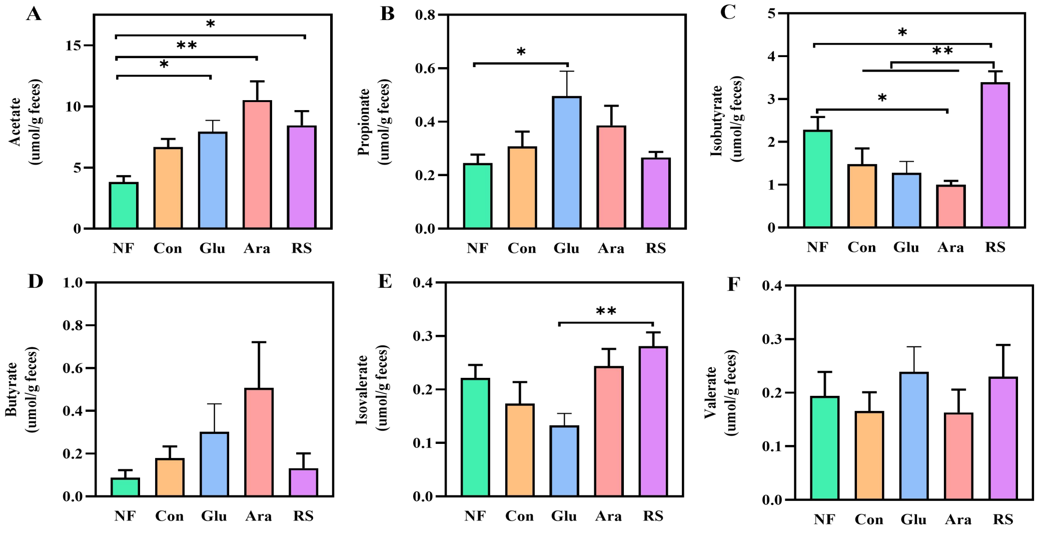

Figure S2. Effect of β-glucan, arabinoxylan or resistant starch supplementation on faeces SCFA. Changes in faece concentrations of (A) acetic acid, (B) propionate acid, (C) isobutyrate acid, (D) butyrate acid, (E) isovalerate acid, (F) valerate acid and (G) total SCFAs. All data are expressed as mean ± SEM (n=10). One-way analysis of variance was performed followed with post-hoc Tukey's test. * means $P < 0.05$, ** means $P < 0.01$. NF, norml fiber levels groups; Con, dietary fiber deprivation group; Glu, β-glucan group; Ara, arabinoxylan group; RS, resistant starch group.

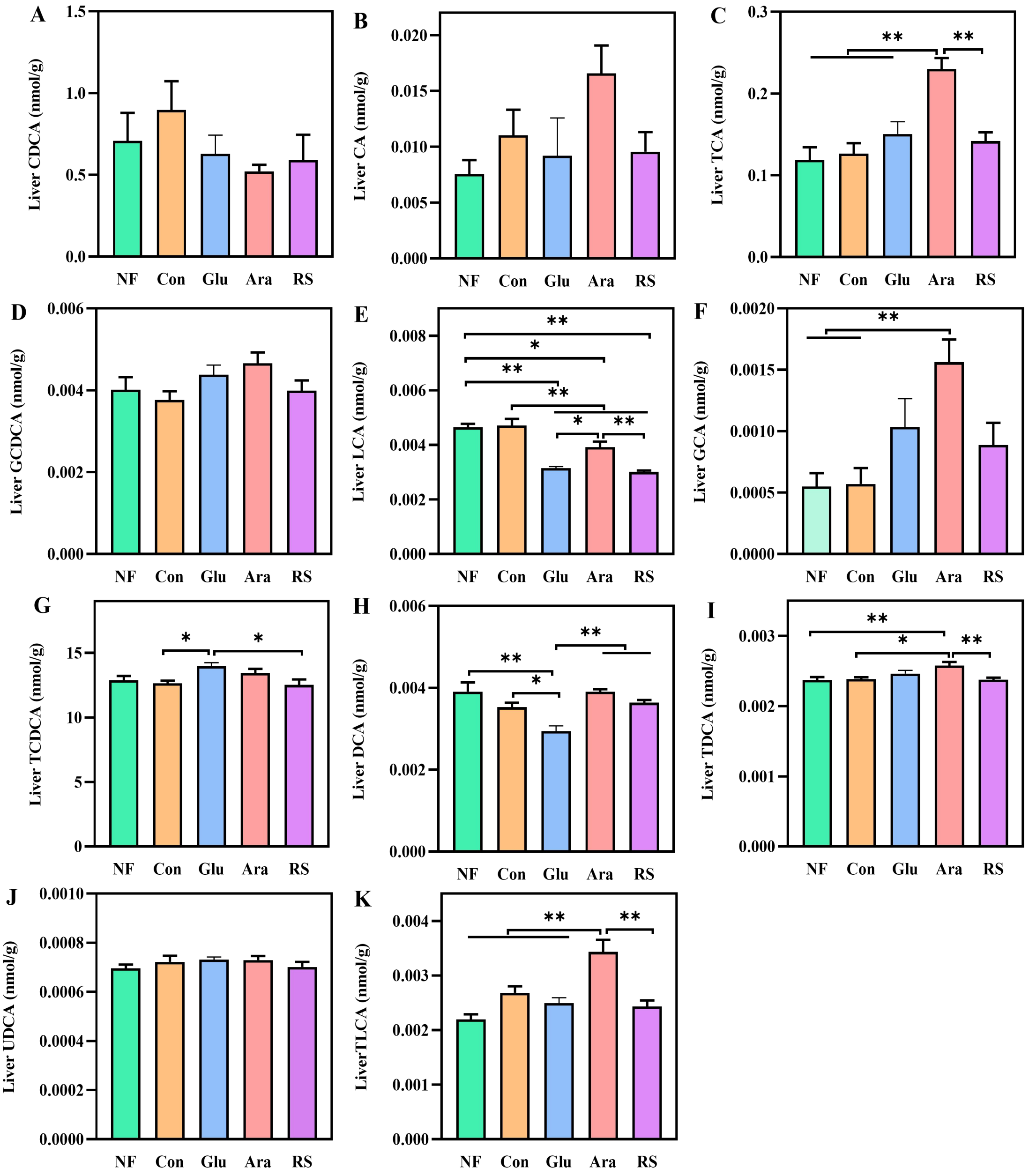

Figure S3. Effect of β-glucan, arabinoxylan or resistant starch supplementation on faeces SCFA. Changes in faece concentrations of (A) acetic acid, (B) propionate acid, (C) isobutyrate acid, (D) butyrate acid, (E) isovalerate acid, (F) valerate acid and (G) total SCFAs. All data are expressed as mean ± SEM (n=10). One-way analysis of variance was performed followed with post-hoc Tukey's test. * means $P < 0.05$, ** means $P < 0.01$. NF, norml fiber levels groups; Con, dietary fiber deprivation group; Glu, β-glucan group; Ara, arabinoxylan group; RS, resistant starch group.
